# Supplementary material for: Approaches for sRNA Analysis of Human RNA-Seq Data: Comparison, Benchmarking
Source: Int J Mol Sci. 2023 Feb 20;24(4):4195. doi: 10.3390/ijms24044195 (PMC9959513; doi:10.3390/ijms24044195)
Supplement: Supplementary file 1 [file ijms-24-04195-s001.zip › Supplemental Captions_17022023.pdf]

Supplemental Figure S1. Read length distribution after trimming procedure across all datasets [58–64].
Supplemental Figure S2. Part of the reads passed the trimming procedure across all datasets [58–64] using all upper bounds.
Supplemental Figure S3. Kallisto alignment rates by kmer length across all datasets [58-64].
Supplemental Figure S4. Salmon alignment rates by kmer length across all datasets [58-64].
Supplemental Figure S5. (A) Distribution of sRNA types expression values by datasets and pipelines (B) Distribution of sRNA types transcripts by datasets and pipelines.
Supplemental Figure S6. Number of transcripts filtered using 6 thresholds (A) by dataset [58-64] and (B) by pipeline.
Supplemental Figure S7. Number of significant differentially expressed transcripts using three DE packages (DESeq2 - top; edgeR - middle; limma - bottom) and two thresholds of significance (adjusted p-value <0.05 - left; p-value<0.05 - right) by two filtering (*mean>5* and *median>5)* and by pipeline.
Supplemental Figure S8. H-score of significant differentially expressed transcripts using three DE packages (DESeq2 - top; edgeR - middle; limma - bottom) and two thresholds of significance (adjusted p-value <0.05 - left; p-value<0.05 - right) by two filtering (*mean>5* and *median>5)* and by pipeline.
Supplemental Figure S9. H-score of significant differentially expressed transcripts using two thresholds of significance (adjusted p-value <0.05 - upper; p-value<0.05 - low) and two filtering (mean>5 - left, median>5 - right*)* by DE package and by pipeline.
Supplemental Figure S10. Distribution of H-scores using three DE packages (DESeq2 - top; edgeR - middle; limma - bottom) and two filtering (mean>5 and median>5) by pipeline groups for all data [58-64].
Supplemental Figure S11. Kallisto alignment rates by dataset [58-64], kmer length and by aligner for tsRNA.
Supplemental Figure S12. Number of reads by dataset [58-64], kmer length and by aligner for tsRNA.

Supplemental Table S1. Alignment rates for alignment-based pipelines across datasets [58-64].
Supplemental Table S2. Assignment rates for alignment-based pipelines across datasets [58-64].
Supplemental Table S3. Assignment rates for pseudoalignment-based methods across datasets [58-64].
Supplemental Table S4. Number of differentially expressed transcripts with adjusted p-value < 0.05 by three DE packages (DESeq2; edgeR; limma) and two filtering (mean>5, median>5*)* across contrasts of datasets [58-64] and pipelines.
Supplemental Table S5. Hobotnica scores for sRNAs by three DE packages (DESeq2; edgeR; limma), two filtering (mean>5, median>5*),* two thresholds of significance (adjusted p-value <0.05; p-value<0.05) across contrasts of datasets [58-64] and pipelines.
Supplemental Table S6. Assignment rates for tsRNA (assigned reads and reads assigned to tRNA ratio) by aligner and kmer length across datasets [58-64].
Supplemental Table S7. Assignment rates for tsRNA (assigned reads and processed reads ratio) by aligner and kmer length across datasets [58-64].
Supplemental Table S8. Number of differentially expressed tsRNA with adjusted p-value < 0.05 by three DE packages (DESeq2; edgeR; limma) and two filtering (mean>5, median>5*)* across contrasts of datasets [58-64] and pipelines.
Supplemental Table S9. Hobotnica scores for tsRNAs by three DE packages (DESeq2; edgeR; limma), two filtering (mean>5, median>5*),* two thresholds of significance (adjusted p-value <0.05; p-value<0.05) across contrasts of datasets [58-64] and pipelines.
Supplemental Table S10. Assignment rates for sRNA-based pipelines across datasets [58-64].
