# Supplementary material for: Approaches for sRNA Analysis of Human RNA-Seq Data: Comparison, Benchmarking
Source: Int J Mol Sci. 2023 Feb 20;24(4):4195. doi: 10.3390/ijms24044195 (PMC9959513; doi:10.3390/ijms24044195)
Supplement: Supplementary file 1 [file ijms-24-04195-s001.zip › Supplemental Figures_17022023.pptx]

## Slide 1
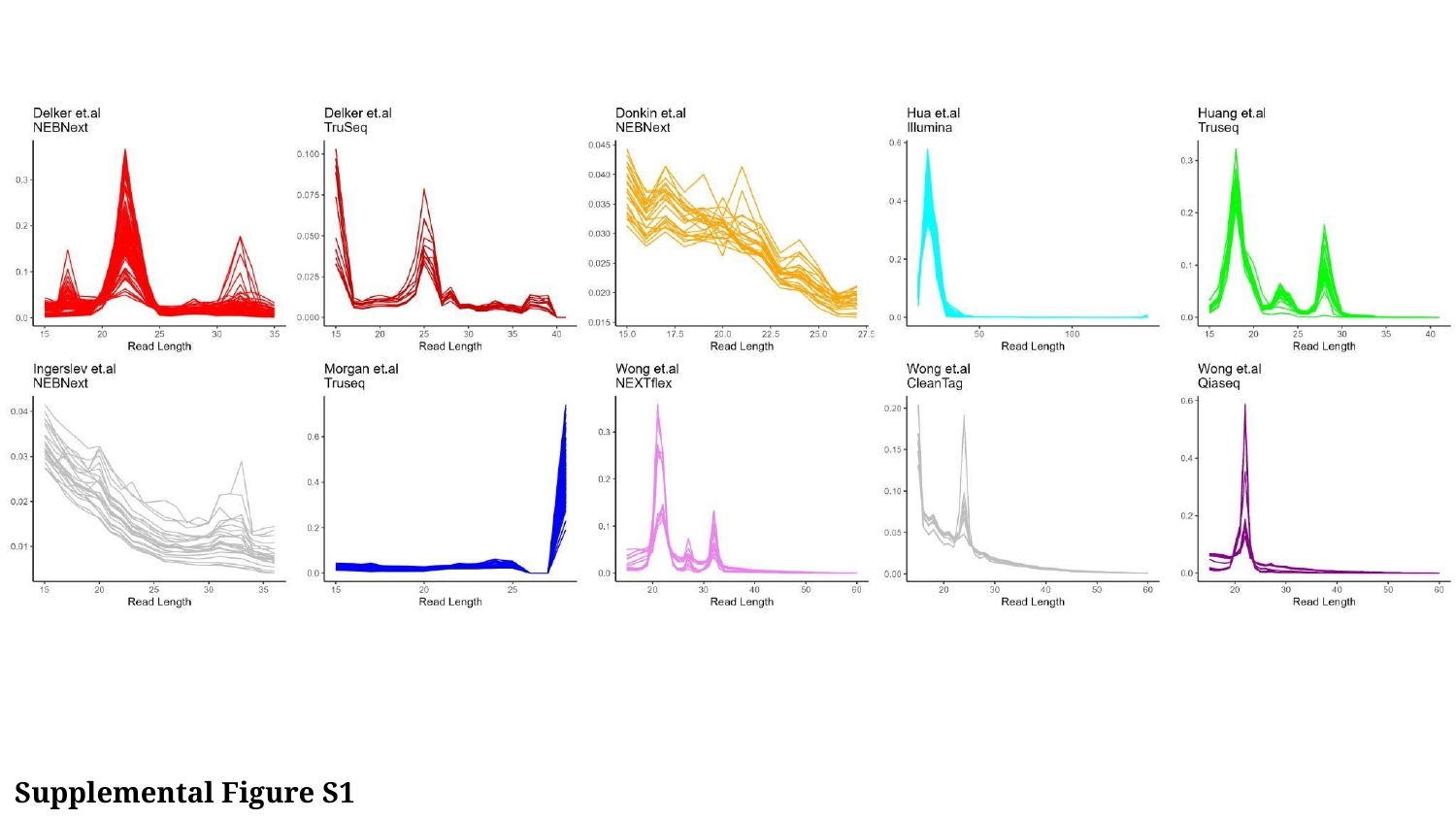

Supplemental Figure S1

## Slide 2
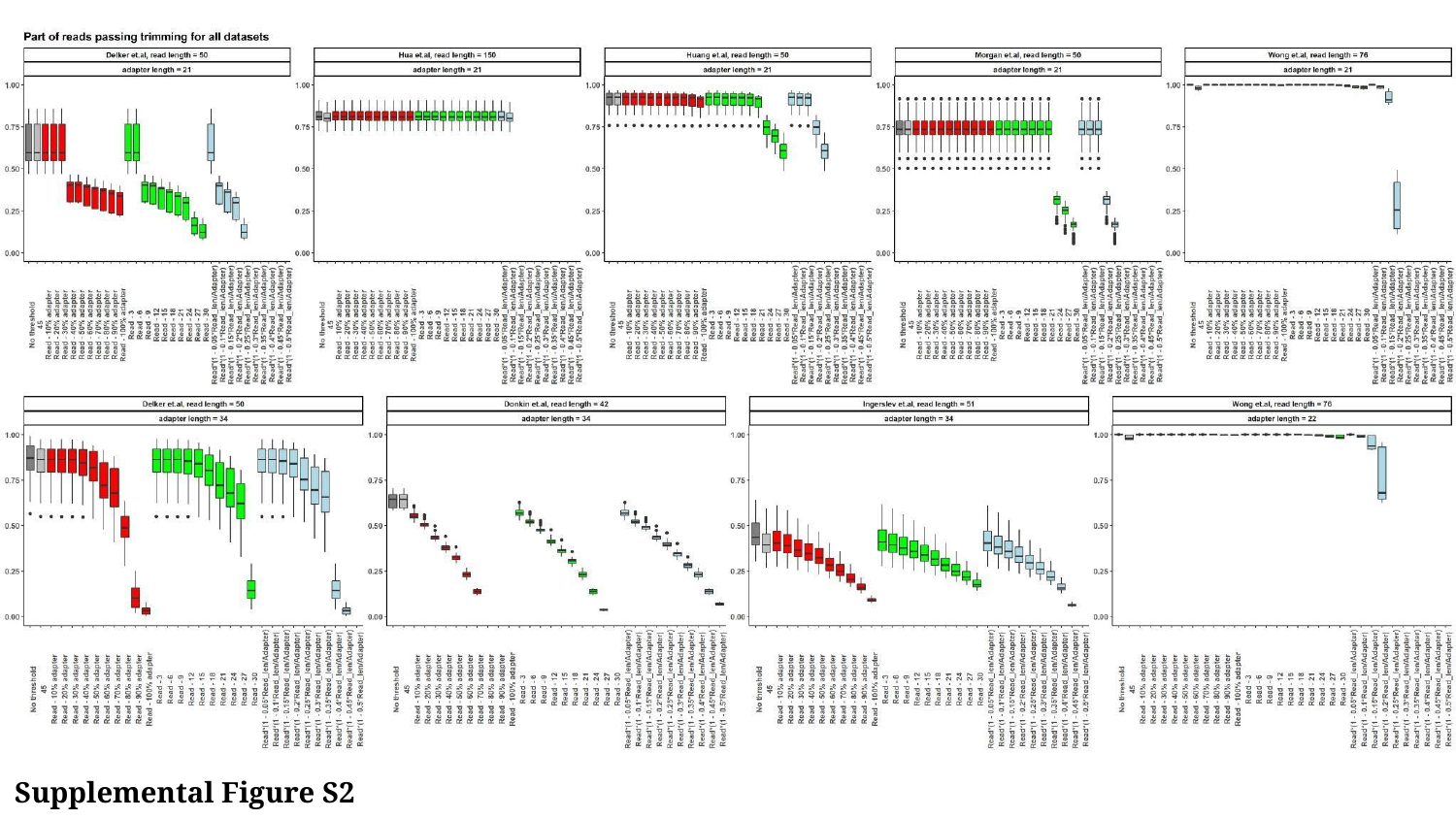

Supplemental Figure S2

## Slide 3
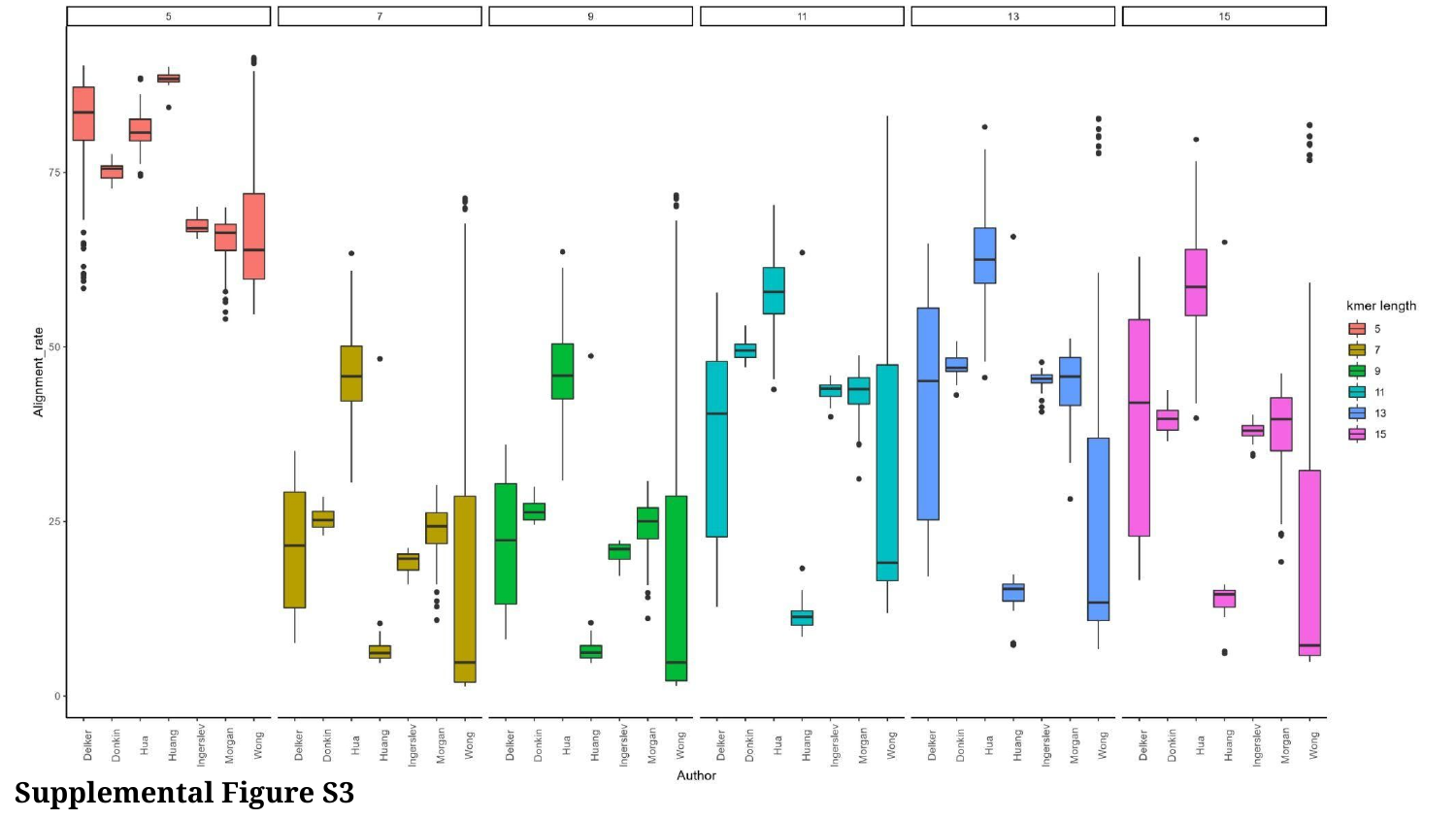

Supplemental Figure S3

## Slide 4
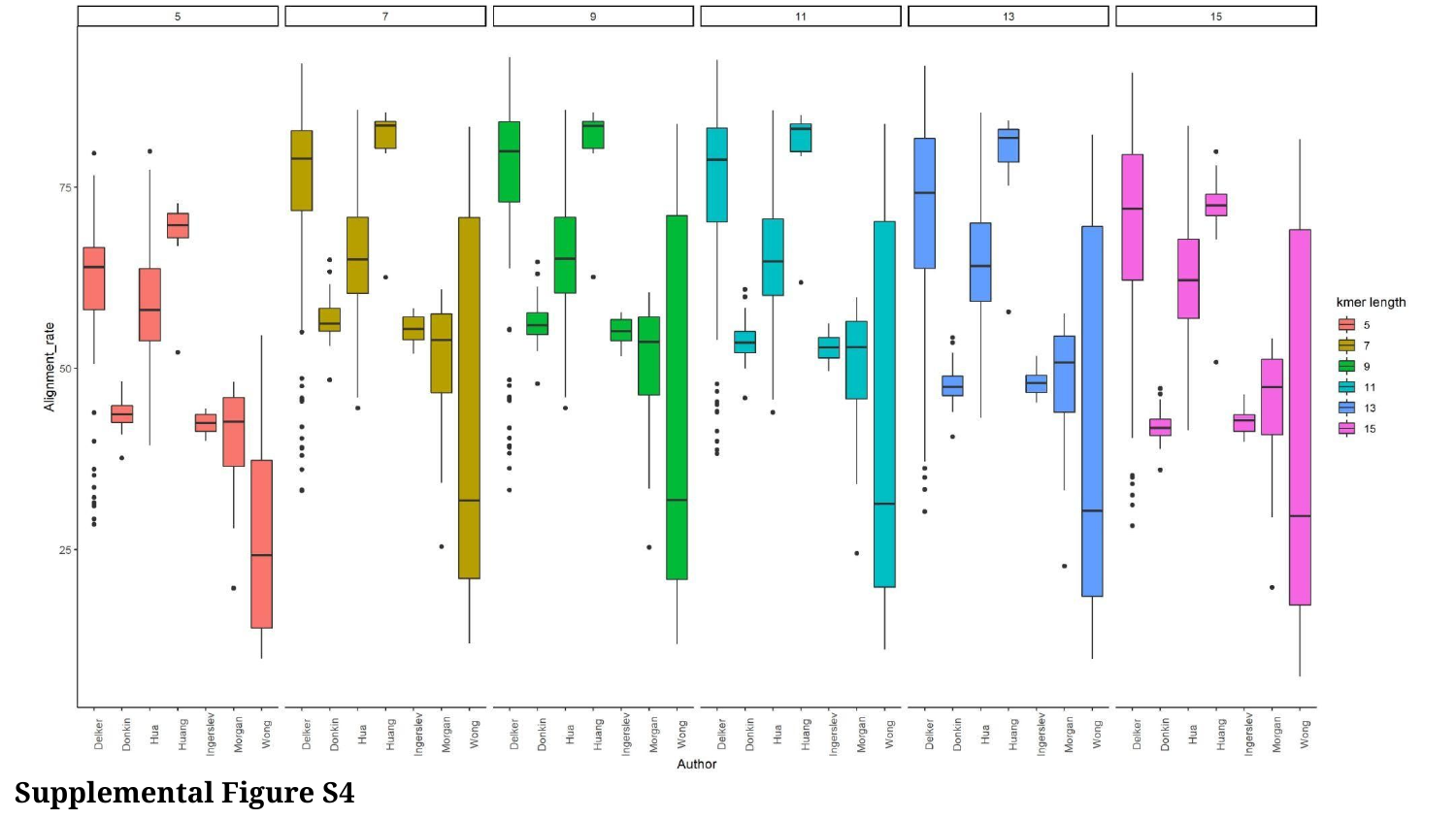

Supplemental Figure S4

## Slide 5
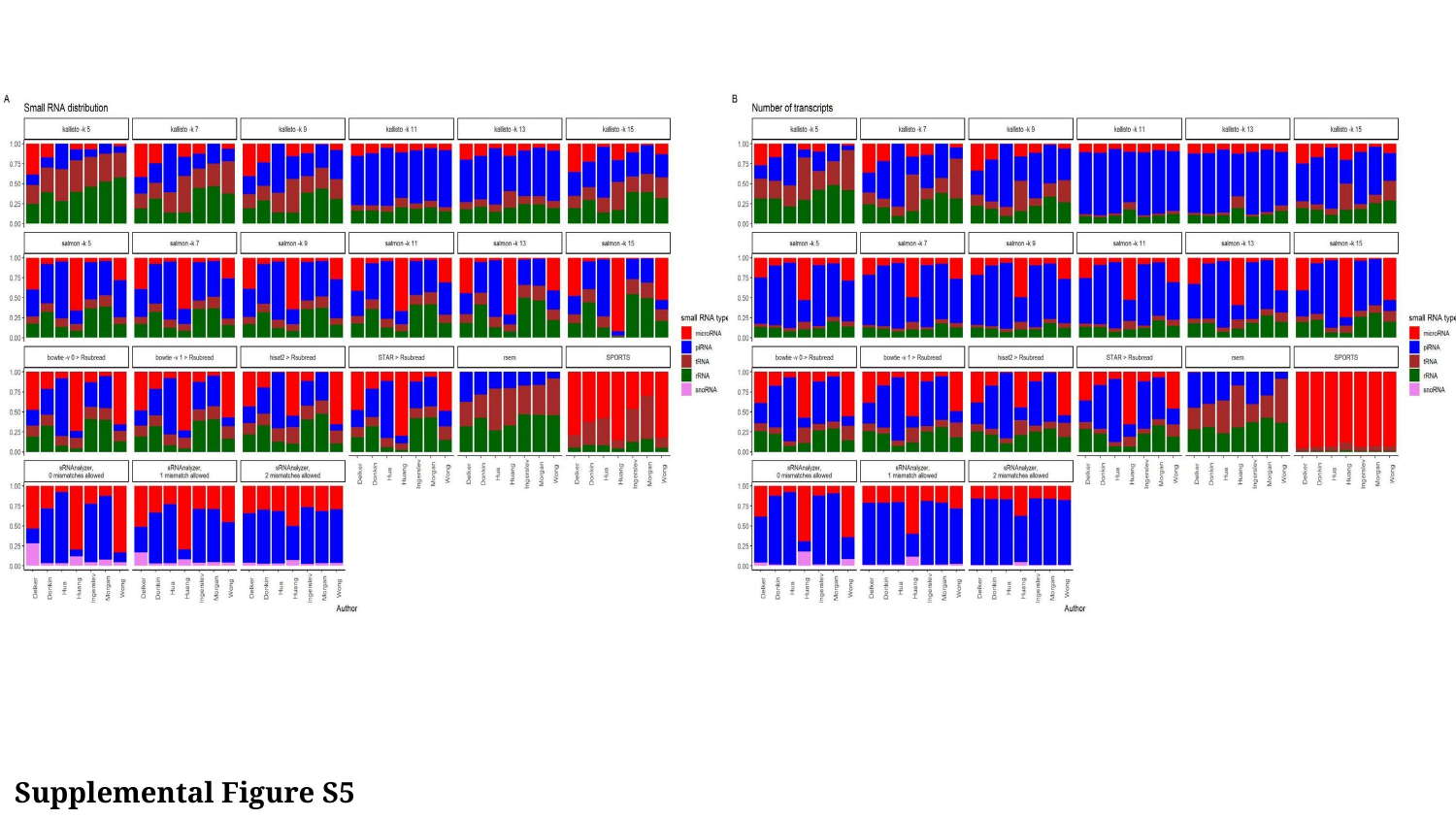

Supplemental Figure S5

## Slide 6
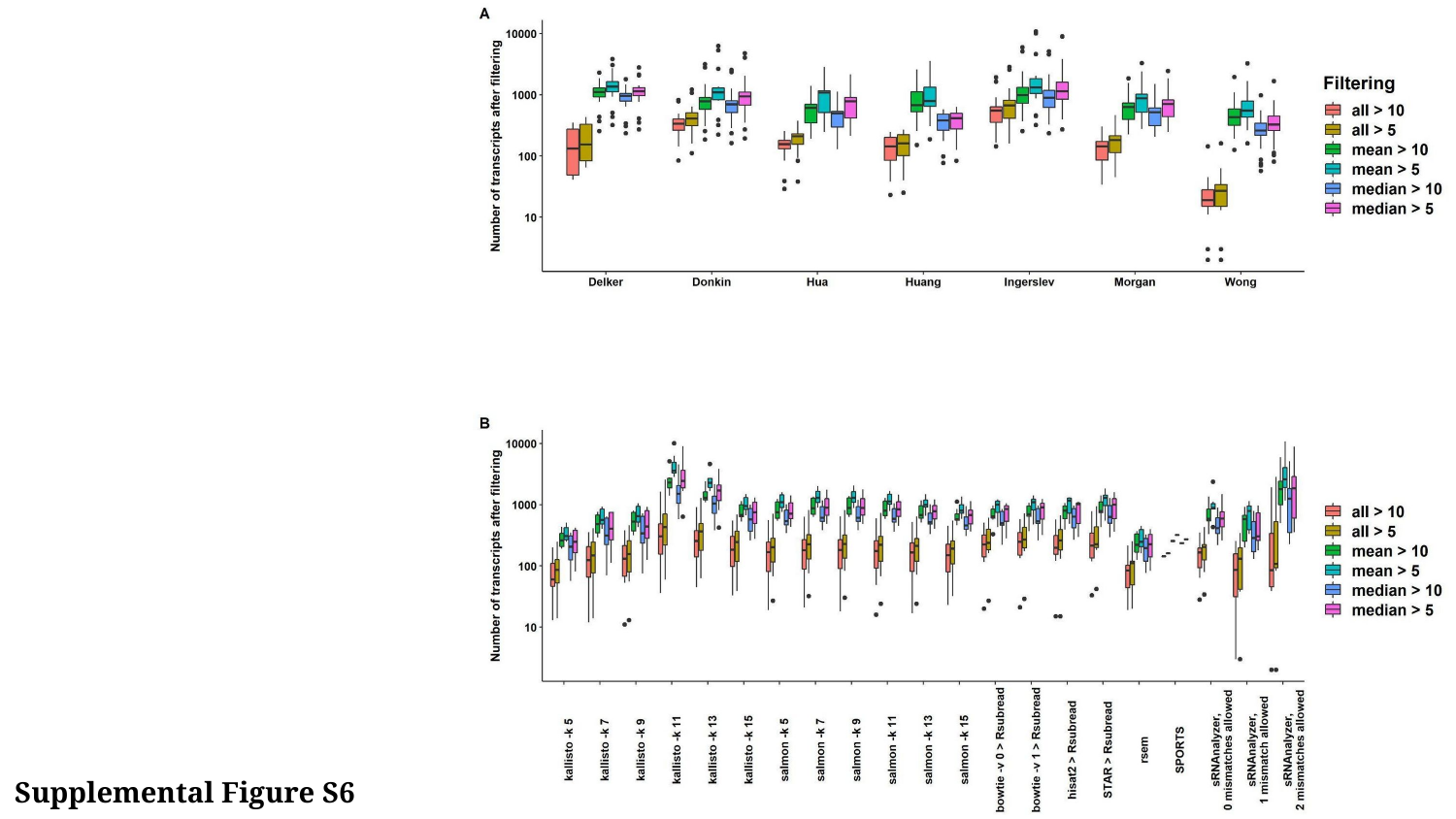

Supplemental Figure S6

## Slide 7
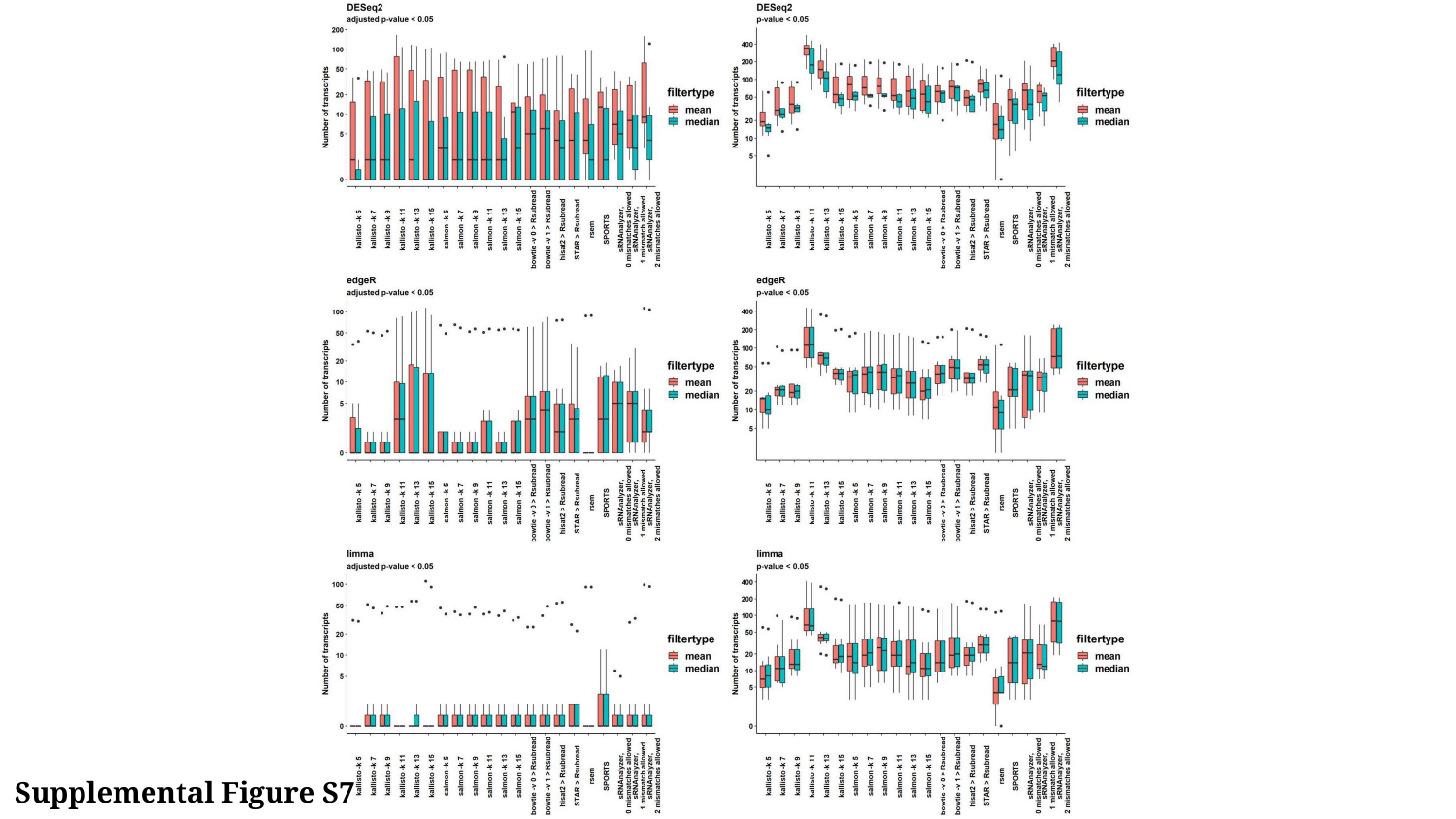

Supplemental Figure S7

## Slide 8
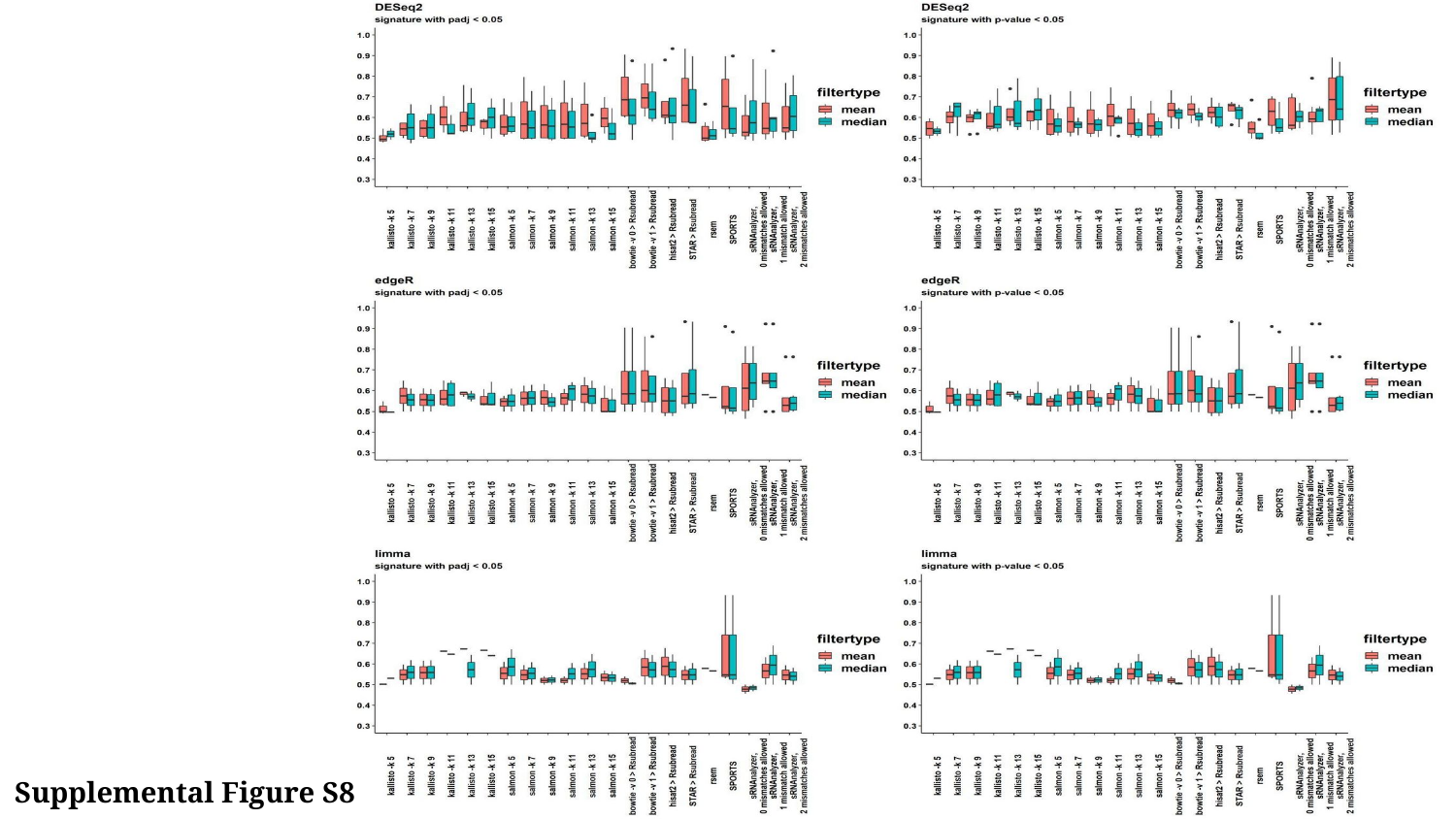

Supplemental Figure S8

## Slide 9
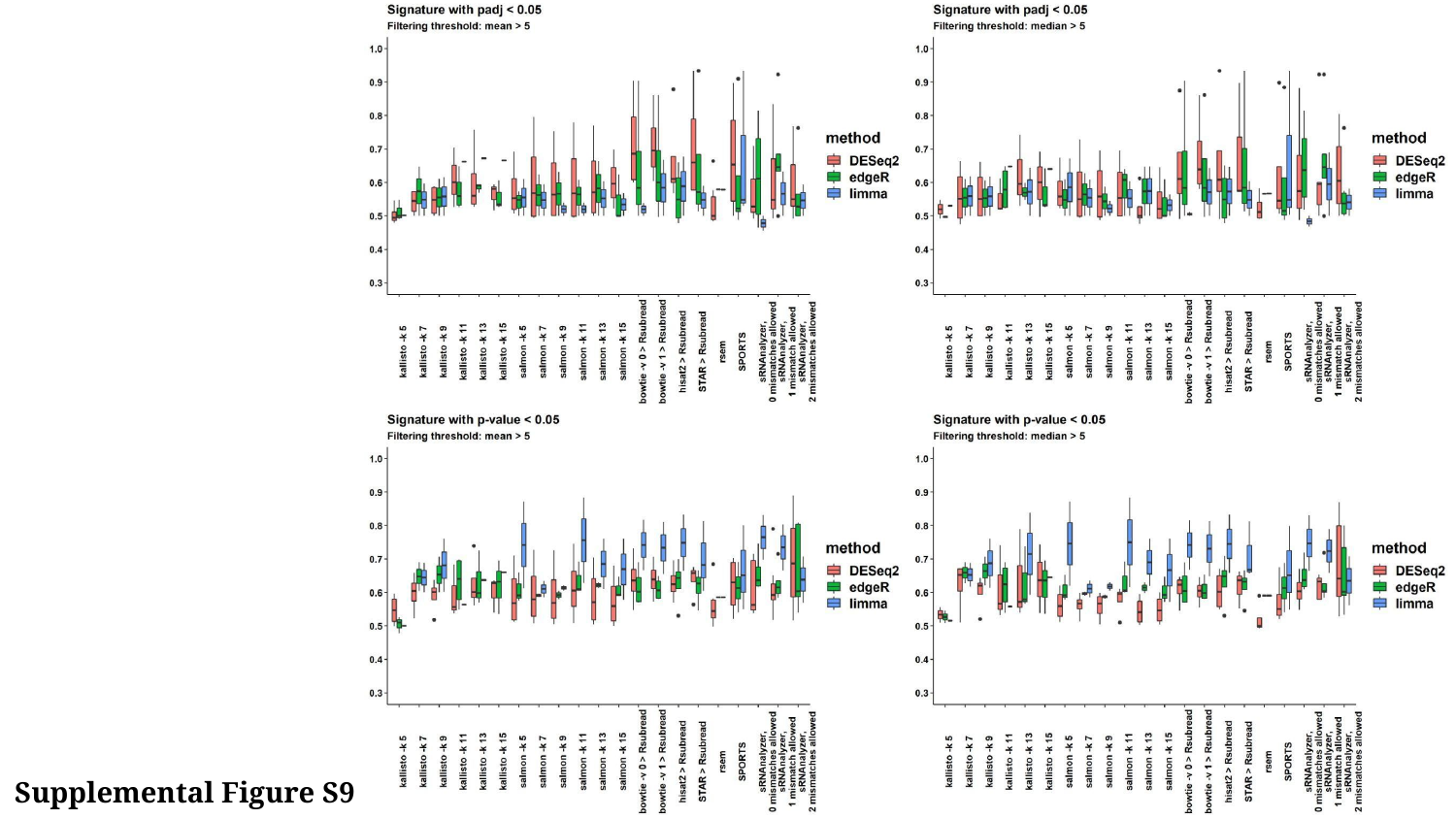

Supplemental Figure S9

## Slide 10
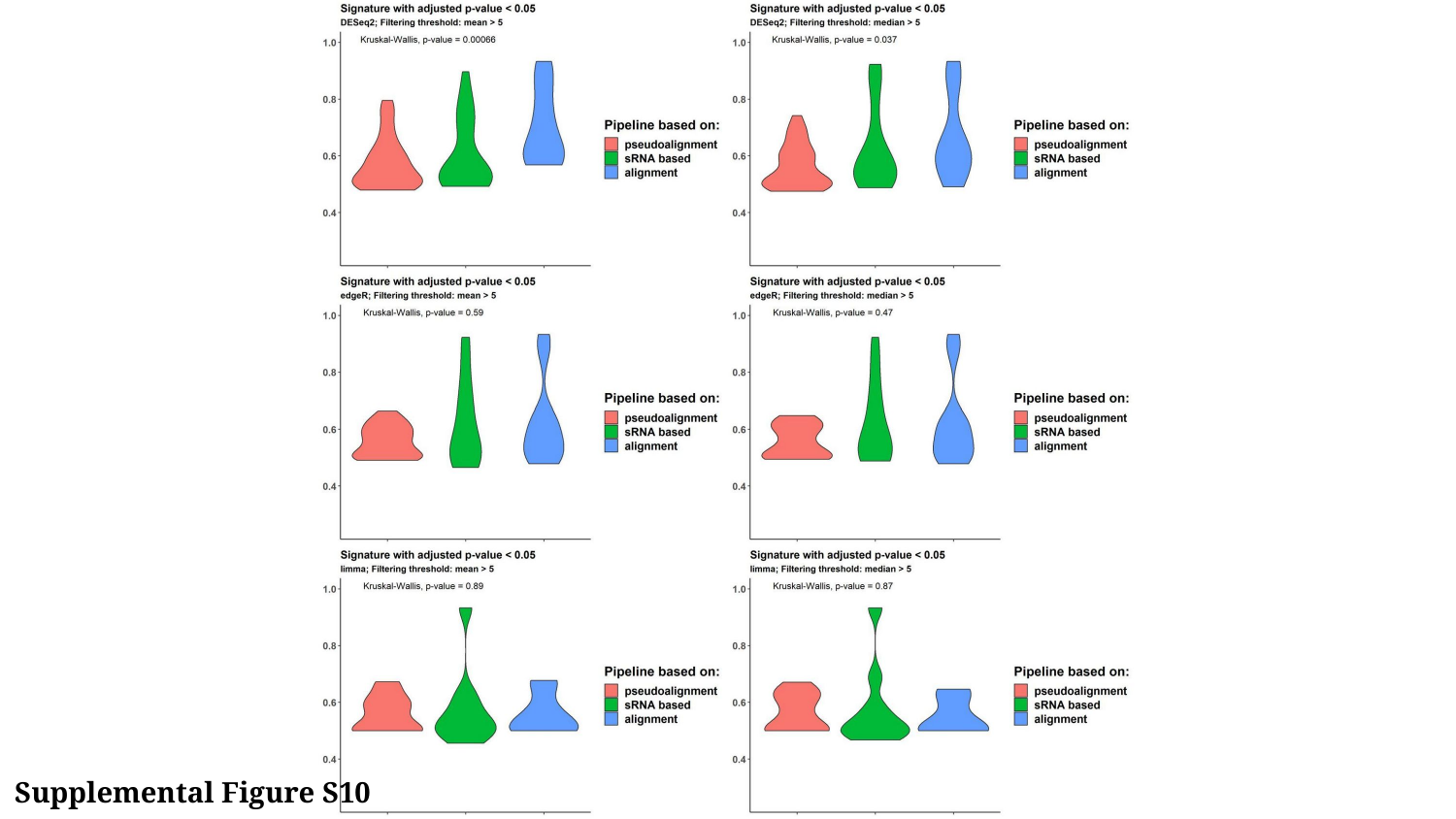

Supplemental Figure S10

## Slide 11
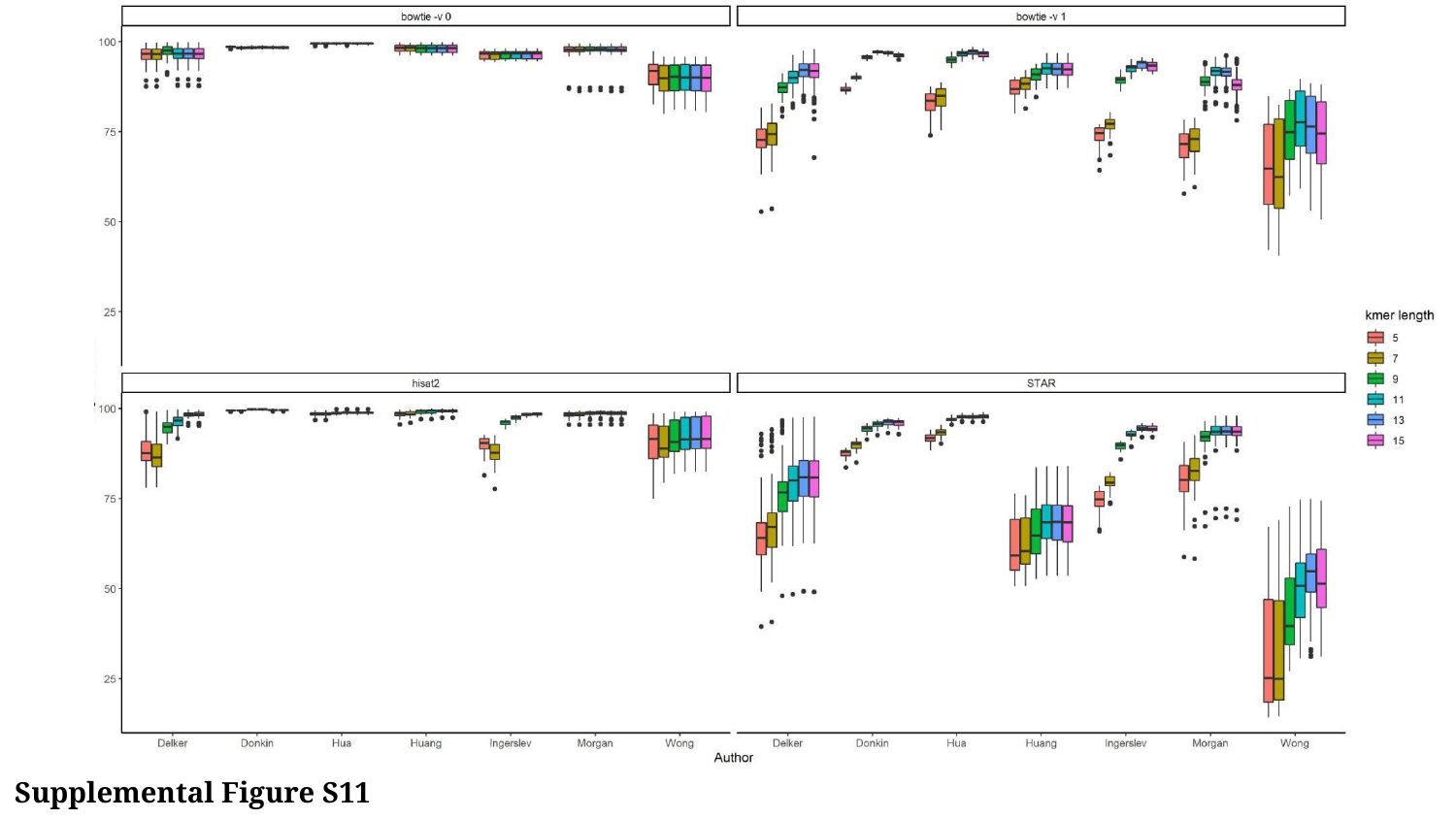

Supplemental Figure S11

## Slide 12
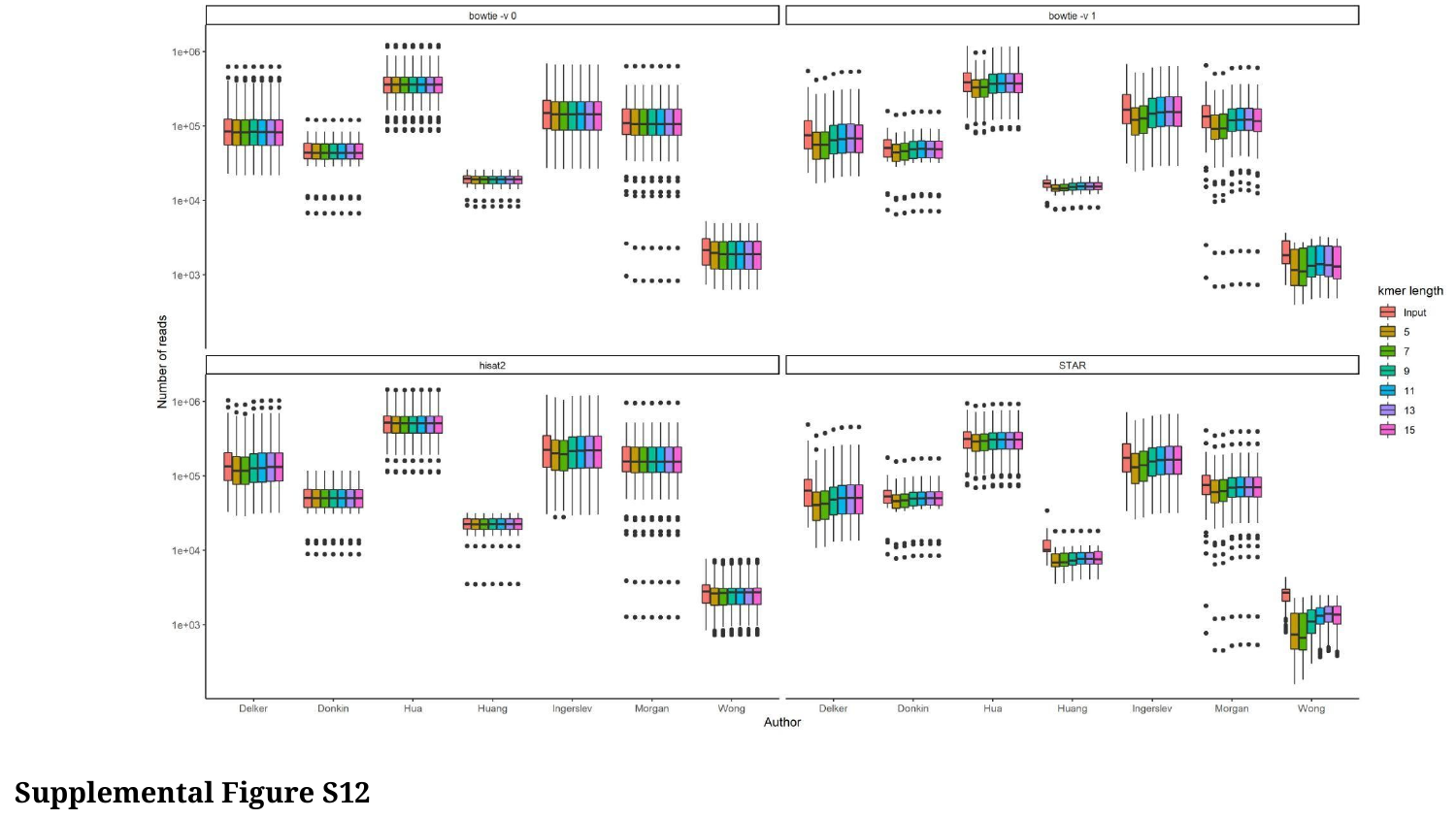

Supplemental Figure S12
